# Characteristics associated with organic food consumption during pregnancy; data from a large cohort of pregnant women in Norway

**DOI:** 10.1186/1471-2458-10-775

**Published:** 2010-12-21

**Authors:** Hanne Torjusen, Anne Lise Brantsæter, Margaretha Haugen, Geir Lieblein, Hein Stigum, Gun Roos, Gerd Holmboe-Ottesen, Helle Margrete Meltzer

**Affiliations:** 1 Division of Environmental Medicine, Norwegian Institute of Public Health, Oslo, Norway; 2 National Institute for Consumer Research (SIFO), Oslo, Norway; 3 Department of Plant and Environmental Sciences, Norwegian University of Life Sciences, Ås, Norway; 4 Division of Epidemiology, Norwegian Institute of Public Health, Oslo, Norway; 5 Department of General Practice and Community Medicine, Faculty of Medicine, University of Oslo, Oslo, Norway

## Abstract

**Background:**

Little is known about the use of organic food during pregnancy. The aim of this study was to describe characteristics associated with the use of organic food among pregnant women participating in the Norwegian Mother and Child Cohort Study (MoBa).

**Methods:**

The present study includes 63,561 women who during the years 2002-2007 answered two questionnaires, a general health questionnaire at gestational week 15 and a food frequency questionnaire at weeks 17-22. We used linear binomial regression with frequent versus rare use of organic food as outcome variable and characteristics of the respondent as independent variables. The outcome variable was derived from self-reported frequency of organic food use in six main food groups (milk/dairy, bread/cereal, eggs, vegetables, fruit and meat).

**Results:**

Organic eggs and vegetables were the food items which were most frequently reported to be used "often" or "mostly". The proportion of women reporting frequent intake of organic food was 9.1% (n = 5754). This group included more women in the lower (<25 years) and higher (>40 years) age-groups, with normal or low body mass index, who were vegetarians, exercised regularly (3+times weekly), consumed alcohol and smoked cigarettes during pregnancy (p < 0.001 for all, except alcohol: p=0.044). Further, participants with frequent organic consumption included more women in the lower (≤12 years) or higher (17 years +) category of educational attainment, women who were students or had a partner being a student, who belonged to the lowest household income group (both respondent and her partner earned <300 000 NOK), who entered the study 2005-2007, and who lived in an urban area (p < 0.001 for all).

**Conclusions:**

The socio-economic characteristics of pregnant Norwegian women with frequent organic consumption did not unambiguously follow those typically associated with better health, such as higher levels of education and income. Rather, lower household income, and both lowest and highest levels of education were associated with a higher prevalence of frequent organic consumption. The results indicate that personal and socio-economic characteristics are important covariates and need to be included in future studies of potential health outcomes related to organic food consumption during pregnancy.

## Background

Dietary quality is especially critical during pregnancy, as adequate nutrition is essential for both maternal and foetal health. There is growing evidence that maternal diet may influence longer-term health of the offspring even within relatively well-nourished populations [1-3]. Dietary intake and food patterns have been described among pregnant women in many populations including Norway [4-6], but little is known about pregnant women who go against mainstream food culture and choose to eat vegetarian diets or organically grown food. In Norway, the generic term for 'organic food' includes food produced at farms following the basic certification requirements as well as farms practicing biological dynamic agriculture. Debio is the Norwegian certification body administering the label 'Ø' for organic food, which is combined with the international label 'Demeter' in the case of bio-dynamic food [7]. The interest for and reported use of organically grown food in the diet has increased in the general public in Norway during the last decade, with women reporting a higher interest than men (25%, as compared to 16%) [8]. It is known from a number of previous studies that health is a main motivation for choosing organic food [9,10]. The expected beneficial health effects of eating organic food during pregnancy include avoiding the possible risks of exposing the developing foetus to synthetic pesticides [11]. On the other hand, higher risks of contamination from mycotoxins, heavy metals and enteric pathogens that could complicate pregnancy have frequently been ascribed to organic production methods, although studies have shown less fusarium mycotoxin contamination [12,13] and lower risk of salmonella and E. coli [14,15] in organic than in conventional foods. Furthermore, higher levels of beneficial substances such as bioactive compounds in plant foods [16,17] and polyunsaturated fatty acids, such as omega-3 and CLA [18] in milk - also found in higher levels in breast milk of lactating women who had eaten organic dairy and/or meat products [19] may be associated with health benefits. A review of literature published in the period from 1958 to 2008 concluded, based on 55 studies, that there is no evidence of a difference in nutrient quality between organically and conventionally produced foodstuffs of relevance to health [20]. Another review by the same authors concluded, based on 12 studies from the same time period, that there is currently no evidence of a health benefit from consuming organic compared to conventionally produced foodstuffs [21]. While the first of these reviews has been criticised for omitting relevant research due to inaccurate selection criteria [22], there seem to be consensus that there is need for further and more detailed studies to investigate possible health effects of eating organic food.

The possible associations between eating organic food and other lifestyle factors and health behavior are not well-described. Information about the prevalence of use of organically grown food within sub-groups of the population is lacking. Such knowledge is important to obtain, especially in critical life phases such as pregnancy, as maternal diet, and possibly also organic food consumption, may have long-term implications for health of the offspring [1,2]. Very few investigations have been conducted concerning the health effects or safety of organic food and no studies have evaluated this in a pregnant human population. The Norwegian Mother and Child Cohort Study (MoBa), a nation wide prospective pregnancy cohort that since 2002 has asked pregnant women about their frequency of organic food use, provides a unique possibility to explore background characteristics and lifestyle behaviors associated with the consumption of organic food during pregnancy. In MoBa we also plan to investigate dietary habits among organic food consumers and to examine potential diet-health relationships related to organic food consumption during pregnancy. To the best of our knowledge, this is the first study to report the use of organic food among pregnant women in relation to a wide range of personal and socio-demographic characteristics.

Compared to other European countries, the development of the organic food market in Norway has been slow [23] and for several decades, organic food had a marginal position outside of the conventional food market [24]. During the last 10-15 years, however, organic products have slowly been introduced into the Norwegian general food market, and sales have grown rapidly in the last few years parallel with an increase in the number of food items certified as organic, particularly since 2005 [25].

Given the close relation between consumers' choice of organic food and their focus on health, as well as the particular relevance of health during pregnancy, the aim of this study was to examine associations between frequent consumption of organic food during pregnancy and health behaviors, socio-economic and demographic characteristics. Could it be that women eating organic food during pregnancy live particularly healthy? Or, on the other hand, could it be that the choice of organic food during pregnancy is associated with lifestyle patterns which are contrary to public advice and knowledge about diet and health relationships?

## Methods

### Subjects and Methods

The data set is part of the MoBa study, initiated by and maintained at the Norwegian Institute of Public Health, and this study is presently the largest pregnancy cohort in the world with 107 000 pregnancies included since 1999 [26]. Pregnant women were recruited to the study by postal invitation after they had signed up for the routine ultrasound examination in their local hospital. The participation rate was about 43% [26]. The women were asked to provide biological samples and to answer questionnaires covering a wide range of information. The cohort database is linked to the Medical Birth Registry of Norway and other national health registries. The MoBa study has been approved by the Regional Committee for Ethics in Medical Research and the Data Inspectorate in Norway.

#### Subjects

This study uses version 4 of the data files made available for research in January 2009. To be included in the present study, the women had to have responded to the general questionnaire at gestational week 15 and to the MoBa food frequency questionnaire (FFQ) at weeks 17-22. In addition, participants had to have answered at least one of the 6 questions about organic food, and have a reported daily energy intake >4.5MJ or <20 MJ [27]. We only used data from the first time participation for women who had been included in the MoBa more than once because of multiple pregnancies. This resulted in a sample of 63,808 women. From this sample, we excluded 250 women who had missing values on age (n = 4) or urban/rural living (n = 246) due to the small numbers, resulting in a final study sample of 63,561.

#### The MoBa food frequency questionnaire

The MoBa FFQ http://www.fhi.no/dokumenter/011fbd699d.pdf is a semi-quantitative food frequency questionnaire designed specifically for assessing diet during the first four months of pregnancy, when the fetus is most vulnerable [3]. The FFQ is described in detail elsewhere [27]. Data has been collected from February 2002 and onwards. The questionnaire has 40 categories of questions covering the daily intake of 255 specific food items and dietary profile such as vegetarian, vegan or partly vegetarian and use of organic food in six main food groups (milk/dairy, bread/cereal, eggs, vegetables, fruit and meat).

#### Outcome variable

The use of organic food was calculated as a sum index based on the question about the frequency of use of organic food specified in six food groups: milk and dairy products, bread and cereal products, eggs, vegetables, fruit and meat. The alternative answers for use of organic food were: 'never or seldom', 'sometimes', 'often', or 'mostly' and were given values from 0-3. For those who had answered at least one of the questions about organic food, missing values for one or more of the other questions were interpreted as 'seldom or never'. The sum index reflects organic food consumption on a scale ranging from 0 to 18, with 0 representing no use of organic food and 18 representing "mostly" organic for all six food groups. Due to inconstancy between the reported dietary profile (vegetarian) and food intakes in the FFQ, we defined 'vegetarian' as having no intake of meat and fish based on the actual food intake. All respondents reported consumption of bread/cereal products and fruit. However, for respondents who had no reported intake of meat (n = 361), eggs (n = 1462), milk/dairy (n = 718) or vegetables (7) and who had not reported organic consumption of the corresponding food group, we upscaled the sum index by multiplying with 6/5 for each omitted food category. This resulted in the upscaling of 817 subjects and included not only vegetarians, but also individuals who avoided relevant food groups due to for instance allergy.

We defined frequent organic consumption as having a sum index of >6, which corresponds to at least one organic food answer in the "often" category. The upscaling resulted in allocation of 48 respondents into the 'frequent organic' consumption group, of which three were vegetarians.

#### Other variables

From the MoBa FFQ, we included the following dietary variables: The inclusion of meat or fish in the diet or a vegetarian diet, and alcohol consumption (yes/no). The variable for vegetarian diet in this study comprises vegans, lacto-vegetarians and lacto-ovo-vegetarians.

From the general questionnaire we included the participant's age, pre-pregnancy body mass index (BMI), smoking, exercise, level of education, the participant or her partner being a student, household income, and year of participation in MoBa. Age was divided into six categories (<20, 20-24, 25-29, 30-34, 35-39, 40+ years). BMI was calculated from self-reported height and pre-pregnant weight and categorized according to the WHO classification as normal (18.5-24.9 kg/m^2^), underweight (≤18.5 kg/m^2^), overweight (25.0-29.9 kg/m^2^), obese grade 1 (30.0 - 34.9 kg/m^2^) or obese grade 2 (≥35.0 kg/m^2^). Smoking during pregnancy was divided into three categories (daily smokers, occasional smokers and non-smokers). Exercise during pregnancy was based on respondents' reports of their participation in 13 different types of recreational exercise and divided into four categories (no exercise, less than once weekly, 1-2 times weekly and 3+ times weekly). Education was divided into three categories: high school or less (≤12 years), 3-4 years of college/university (13-16 years) or four or more years of college/university education (17+ years). Being a student (yes/no) was included for both the participant and her partner. Household income was measured as a combination of the participant's and her partner's income (both <300 000 NOK, one ≥300 000 NOK, or both ≥300 000 NOK). Urban or rural living area was explored using several variables: 1) an urban/rural variable based on 35 selected urban municipalities with >20,000 inhabitants and city status, 2) a variable based on 32 selected rural municipalities with a special focus on organic food, and 3) a variable indicating geographic location of a municipality in relation to urban settlements of various sizes, according to definitions obtained from Statistics Norway [28].

### Statistical analyses

We used polychoric correlation to examine organic consumption among all food groups. The differences in organic food consumption between categories of maternal characteristics were tested using chi-square, and p value of <0.05 were considered to indicate statistical significance. We used linear binomial regression, with frequent use of organic food (sum index >6) as the binomial outcome variable. This analysis provides information about the risk difference (RD) between being in the frequent organic intake group or not, given various personal, socio-economic and lifestyle characteristics as covariates. Linear rather than logistic regression was preferred because the risk estimates for various constellations of covariates may be directly interpreted from the adjusted model as prevalence at reference category +/- the risk difference for any given characteristic/covariate, e.g. year 2005, age 35-39 etc. The model was checked by repeating the analysis using linear regression with the sum index as a continuous outcome variable, and comparable results were obtained (not shown).

A total of 13,174 (20.7%) women had missing values on one or more of the covariates. Participants with missing data on a variable are often categorized in a "missing" category to avoid exclusion of incomplete cases. This may, however, introduce bias. We therefore performed the linear binomial analysis in the full sample (n = 63,561) as well as in complete cases only (50,387) to examine the influence of including incomplete cases. The results were almost identical and we chose to present results for the full sample.

All analyses were performed using the statistical software PASW statistics 17 (SPSS Inc., IBM Company, Chicago, Ill., USA), except the polychoric correlation analysis which was performed in Stata version 11 (Stata Corp, Texas 77845 USA).

## Results

Eggs and vegetables were the food categories that most respondents reported to use 'often' or 'mostly' organic, while few women reported consumption of organic meat. Across all food categories, the 'never/seldom' organic was the dominant answer, ranging from 65.4% for vegetables to 88.7% for meat, while the most frequent organic user group was small, ranging from 1.0% for meat to 3.5% for eggs. A total of 3376 (6.7%) women reported 'mostly' organic consumption for at least one of the six food groups (Table 1). There was a high correlation between organic food consumption across the food groups, with vegetables and fruits being most strongly correlated with each other and with the sum index (Table 2).

**Table 1:** Self reported organic consumption of six main food categories§.

	'Never/seldom' (Value 0) % answered	'Sometimes' (Value 1) % answered	'Often' (Value 2) % answered	'Mostly' (Value 3) % answered
Milk/dairy	74.5	18.3	4.6	2.5
Bread/cereal	80.0	13.7	3.7	2.6
Eggs	66.2	24.2	5.7	3.4
Vegetables	65.4	27.2	5.6	1.7
Fruit	71.7	21.7	4.7	1.8
Meat	88.0	7.8	3.1	1.1
Any	92.8	44.9	13.6	7.0

^§^Percentage reported use within each food category. N = 63,561 pregnant women in the Norwegian Mother and Child Cohort Study 2002-2007

**Table 2:** Polychoric correlation between the reported use of organic food categories (n = 63,561)

	Milk/ dairy	Bread/ cereal	Eggs	Vegetables	Fruits	Meat	Sum **index**^**§**^
Milk/dairy	1	0.79	0.68	0.73	0.72	0.74	0.76
Bread/cereal		1	0.65	0.75	0.79	0.81	0.78
Eggs			1	0.74	0.70	0.65	0.73
Vegetables				1	0.92	0.78	0.82
Fruit					1	0.82	0.82
Meat						1	0.73

^§^Sum index is the summation of reported use across all food categories

When summing the reported use of organic food across the six food groups, 1.9% of the women reached a sum index of 13 to 18, 7.2% had a sum index of 7 to 12, 39.2% had a sum index of 1 to 6 and 51.7% a sum index of 0, indicating that they had answered never or seldom to all organic food categories. In the further analysis we chose to define frequent organic consumption as having a sum index >6, which corresponds to reported consumption of at least one organic food group in the 'often' category. This resulted in 5754 (9.1%) frequent organic consumers (Table 3). There was a strong effect of age on frequent organic food consumption; among 30-34 year old there were 8.1% frequent consumers, compared to 28% among <20 year old, a crude difference of 20 percentage points (pp). The adjusted difference from the model was 15.6 pp (Table 3).

**Table 3:** Association between socio-economic, personal and lifestyle factors and frequent consumption of organic food.

		Unadjusted model			**Adjusted model**^**§**^	
		SumIndex >6		P-value*	RD*100	(95% CI)
	Total n 63,561	n	%			
Total			9.1			
Prevalence at ref. category					7.8	
Age				*<0.001*		
<20	854	239	28		**15.6**	**(12.6,18.7)**
20-24	7745	996	12.9		**3.0**	**(2.1, 3.8)**
25-29	22689	1820	8.0		-0.5	(0.9, 0.0)
30-34	22925	1866	8.1		0	
35-39	8306	715	8.6		0.3	(-0.4, 0.9)
40 +	1042	118	11.3		**2.6**	**(0.7, 4.5)**
Prepregnant BMI				*<0.001*		
<18.5	1825	226	12.4		**1.8**	**(0.4, 3.3)**
18.5-24.9	40519	3795	9.4		0	
25-29.9	13547	1102	8.1		**-0.9**	**(-1.4, -0.4)**
30-34.9	4355	314	7.2		**-1.8**	**(-2.5, -1.1)**
35+	1645	128	7.8		**-1.5**	**(-2.7, -0.3)**
Missing	1670	189	11.3		**1.0**	(-0.5, 2.5)
Dietary habits				*<0.001*		
Meat/fish	63449	5716	9.0		0	
Vegetarian	112	38	33.9		**23.1**	**(14.4, 31.8)**
Alcohol in pregnancy				*0.59*		
No	56182	5072	9.0		0	
Yes	7379	682	9.2		**0.7**	**(0.0, 1.4)**
Smoking in pregnancy				*<0.001*		
No smoking	58241	5116	8.8		0	
Occasionally	1812	218	12.0		**2.0**	**(0.6, 3.4)**
Daily	3508	420	12.0		**1.5**	**(0.5, 2.6)**
Exercise in pregnancy				*<0.001*		
No	9166	756	8.2		0	
Less than weekly	12317	888	7.2		-0.5	(-1.1, 0.2)
1-2 times weekly	18734	1538	8.2		0.4	(-0.3, 1.0)
3+ times weekly	17992	1956	10.9		**2.5**	**(1.8, 3.2)**
Missing	5352	616	11.5		**2.6**	**(1.7, 3.6)**
Education				*<0.001*		
<10 y-12 y	20398	2252	11.0		**0**	
13-16 y	26462	1822	6.9		**-2.5**	**(-3.0, -1.9)**
17+	15319	1534	10.0		-0.1	(-0.7, 0.6)
Missing	1382	146	10.6		-0.2	(-1.8, 1.4)
Student, participant				*<0.001*		
No	57279	4928	8.6		0	
Yes	6282	826	13.1		**1.9**	**(1.1, 2.8)**
Student, participant's partner				*<0.001*		
No	60296	5342	8.9		0	
Yes	3265	412	12.6		**1.7**	**(0.6, 2.9)**
Household income				*<0.001*		
Low (both <NOK 300 000)	17324	1861	10.7		0	
Medium (one ≥NOK 300 000)	24357	1947	8.0		**-1.6**	**(-2.1, -1.0)**
High (both ≥NOK 300 000)	16561	1268	7.7		**-2.3**	**(-2.9, -1.6)**
Missing	5319	677	12.7		**1.0**	(-0.0, 1.9)
Year of participation				*<0.001*		
2002	8768	679	7.7		0	
2003	10834	855	7.9		0.3	(-0.4, 1.0)
2004	10717	834	7.8		0.4	(-0.3, 1.1)
2005	12369	1135	9.2		**1.5**	**(0.8, 2.2)**
2006	11030	1097	9.9		**2.3**	**(1.6, 3.1)**
2007	8562	993	11.6		**4.1**	**(3.3, 5.0)**
Missing	1281	161	12.6		**3.6**	**(1.7, 5.4)**
Living area				*<0.001*		
Rural	31070	2675	8.6		0	
Urban	32491	3079	9.5		0.6	**(0.1, 1.0)**

^§ ^Linear binomial model. Prevalence at reference category shows the constant term which equals expected use of organic food with medium or high frequency (Sum index >6) when all covariates are zero.

The table shows risk differences × 100 (with 95% confidence interval) for frequent consumption of organic food. Zero values are the reference categories.

*P for trend across characteristic categories (Chi square).

Higher prevalence of frequent organic use was also found among women in the underweight and normal weight BMI categories. The largest difference in use of organic food was found for eating a vegetarian diet (RD = 23.1 pp.), while only a very low share of participants were vegetarians (0.2%) (Table 3).

Overall, 91.6% of the women in this study were non smokers and 88.4% abandoned alcohol completely during pregnancy. However, among smokers and among women who consumed alcohol during pregnancy, there was a higher prevalence of frequent organic use. Further, physical activity three times a week or more was associated with a higher prevalence of organic food consumption (Table 3).

With regard to education, there was a two-sided trend with more frequent organic food consumption among those with 12 years or less or 17 years or more, while it was lower in the middle category. There was a higher likelihood of frequent organic consumption in households where the participant and/or her partner were students, and in households with low income. The likelihood of frequent organic consumption was in fact inversely correlated with higher income (Table 3).

Participation in MoBa at later years (2005 or later) was associated with frequent use of organic food. This was also the case for those living in urban areas. When using a four-level variable for living area, based on traveling distance to nearest town or city, the largest share of women with frequent use of organic food was found in the most central areas (9.4%), followed by the least central municipalities (8.6%). The lowest prevalence of frequent organic consumption was in the middle-category municipalities (8.0% and 8.4% in less or quite central municipalities respectively) (data not shown in table).

By combining the risk differences for the variables in the model we theoretically estimated that the group with the highest propensity to have a frequent intake of organic food were women <20 years of age, having BMI < 18.5, adhering to a vegetarian diet, consuming alcohol and smoking occasionally during pregnancy, exercising three times a week or more, having attained less than 13 years of education, being a student and having a partner who is a student, having low household income (both the participant and her partner earned <300 000 NOK), participating in MoBa in year 2007, and living in an urban area. The estimated prevalence of a frequent intake of organic food for this group was 61.8% (prevalence at reference category + risk difference for the relevant values of all the variables in the model). In the same way we estimated the prevalence in the group with the lowest propensity to have frequent intake of organic food to be 0.2%.

## Discussion

The main finding of this study was that no single "healthy lifestyle" orientation could be identified among the women who reported frequent use of organic food. The frequent use of organic food was associated with lower and higher age groups, lower BMI, a vegetarian diet, cigarette smoking and use of alcohol during pregnancy, regular exercise, lower and higher levels of education, the participant and/or her partner being a student, low household income, urban living area, and participation in MoBa between 2005 and 2007. The associations between socio-demographic and lifestyle variables and eating organic food reflect complexity and indicate that no quick label like "young and idealistic" or "well educated and wealthy" can be applied to describe women who report frequent intake of organic food during pregnancy. Some previous studies also reported that use of organic food is quite widely distributed across socio-economic groups and associated with various types of motivation [10,29], while others, particularly within the marketing tradition, have identified consumer segments in the market with a high likelihood to buy organic food such as "the engaged", "the eco-healthy", or "the practical green" etc. [30].

The strength of this study is the large sample of pregnant women with participants from both urban and rural regions, representing all age groups and all socioeconomic groups. The participation rate in MoBa is 43% and the prevalence of organic consumption may not be representative for all pregnant women in Norway [26]. However, this is not likely to influence the associations between reported use of organic food and characteristics of the respondents. The potential bias due to self-selection in MoBa was recently evaluated by Nilsen et al., 2009. No statistically relative differences in association measures were found between participants and the total population regarding eight exposure-outcome associations evaluated [31].

In the present study we examined the associations between participant characteristics and frequent organic consumption with and without women having missing data on participant characteristics. Covariates with missing data were BMI (2.6%), exercise (8.4%), education (2.2%), income (8.4%), and year of participation (2.0%). The numbers of missing values were higher in year 2002, in the youngest age group, among women with low education, among smokers, and among frequent organic food consumers. Including missing as separate categories in a regression is a simple method of dealing with missing values compared to the more correct but also more complex method of multiple imputations. Including missing in a regression will, contrary to popular belief, increase bias from confounding, but will reduce bias from possible heterogeneity of effects (interaction) between responders and non-responders/missing. It will also increase sample size and thereby power. The results from models with and without missing included were similar in our data, indicating that neither confounding nor heterogeneous effects played a strong role here.

The sum index provides a robust indicator of the consumption of a variety of the main organic food groups in the diet, appropriate for the explorative aim in this study. The sum index attributes equal importance to each food category and thus some detail may have been lost. The categories are dissimilar with regard to number of items within the category (e.g. 'eggs' containing only one item while 'fruit' and 'vegetables' contain numerous items). Eating 'mostly organic' vegetables is a more extensive practice than eating 'mostly organic' eggs. A related, but different challenge is that we do not know a respondents total variety of consumption within each category, e.g. whether 'mostly organic fruit' refers to only apples - or a whole range of different fruits. However, the high correlation between organic consumption within the six food groups supports the viability of using a sum index in this study (Table 2).

Eating a vegetarian diet was the characteristic which was most strongly associated with frequent consumption of organic food during pregnancy. Even though the total number of vegetarians among the MoBa-participants was low (0.2%), this was the single most predictive factor among all variables in the analysis, with 23.1 percentage points higher prevalence of frequent organic use among vegetarians than among non-vegetarians (Table 3). This reinforces earlier findings of an association between eating organic food and a vegetarian diet [32,33]. Frequent use of organic food and eating a vegetarian diet may well be part of a healthy lifestyle, as a vegetarian diet has been associated with many health benefits such as lower risk of heart disease and type 2 diabetes [34-37]. Further associations between eating a vegetarian diet and the general dietary quality among the women with a frequent consumption of organic food will be published in a separate paper. Well-planned vegetarian diets are considered appropriate for individuals in all life-phases, including pregnancy [34]. Lower levels of BMI have been reported among vegetarians in various populations [35,37], and also among consumers of organic food in a study of soy consumers and non-soy consumers in Minnesota, USA [38].

Participation in regular exercise and being underweight or normal weight were also associated with being a frequent organic consumer. Being physically active is an important contributor to a healthy lifestyle in the general population as well as among pregnant women, and is strongly inversely related to excessive body mass [39-41].

Entering pregnancy with a normal weight is beneficial with regard to pregnancy complications and health outcomes for both the mother and the child. Maternal obesity is a risk factor for all major pregnancy complications, which have increased in prevalence in later years [42], including gestational diabetes, pre-eclampsia, foetal overgrowth, preterm births, and cesarean delivery [42-45]. Being underweight, on the other hand, is also associated with unfavorable birth outcomes such as preterm birth and low birth weight, while overall, the outcome is favorable and several adverse outcomes are less common in this group of women [46]. We plan to further investigate dietary habits among organic food consumers and to examine potential diet-health relationships related to organic food consumption during pregnancy. It would also be interesting to further investigate subgroups within the population, as the characteristics associated with frequent organic consumption in the present study have also been related to a higher prevalence of eating disorders, such as adhering to a vegetarian diet [47], exercising more than three times per week, older age and being a student [48].

It is well established that both cigarette smoking and alcohol consumption during pregnancy is associated with increased risk of adverse health outcomes for the fetus, and consequently health authorities in many countries, including Norway recommend that pregnant women and those trying for a baby should totally avoid alcohol and smoking [49]. In this study we found a higher prevalence of smokers among women with frequent consumption of organic food. If the use of organic food is motivated by perceived health benefits, this finding may appear surprising. However, sociological studies of health behavior have indicated that the associations between them are complex. In a Finish study, an attempt to construct 'health indices' based on all relevant factors associated with good health proved difficult, and even though there were clear associations between health behaviors, their distribution into different combinations were quite diverse [50,51]. Smoking has been shown to be central in the interplay between health behaviors, and the majority of smokers had either only smoked or had one additional unhealthy habit [51].

Our finding of a higher prevalence of cigarette smoking among women with a frequent consumption of organic food is contrary to a European multi-country study that reported less maternal smoking during pregnancy and current smoking in families with anthroposophic lifestyle (having children at Waldorf schools, eating organic/bio-dynamic food and/or living at farms practicing Bio-dynamic farming) compared to reference families [52,53], while a Swedish study reported equal prevalence of parental smoking in families with anthroposophic lifestyle vs. reference families [54]. In the present study, however, consumers of all types of organic food are included, and we have not looked at respondents adhering to an anthroposophic lifestyle in particular.

We found a two-sided trend between frequent use of organic food both in regard to age and education, with the highest and lowest age and education groups being more likely to be frequent organic consumers than the middle groups. Next to eating a vegetarian diet, being in the lowest age group (<20 years) represented the highest likelihood for being a frequent organic consumer, with 15.6 percentage points higher prevalence than in the reference group (30-34 years). The present study has a smaller age range than studies in the general population as only pregnant women were included. Studies in the general population have also reported diverging results related to age, some finding that younger age (see for example [55-58]) and others that older age [59] was associated with a higher propensity to buy organic food, while some reported no difference with regard to age [29]. It has been suggested that there may be a pattern whereby there are higher shares of younger consumers among early adopters in developing markets, while older consumers are in higher numbers in more mature markets [60]. In Denmark and Great Britain, both being mature markets for organic food, the highest likelihood of buying organic food was found in households with middle-aged. In Great Britain the likelihood was lower in both the younger and older groups, while in Denmark it generally increased with age, but with a peak for the age group 40-49 years [61]. The Norwegian market for organic food is not by far as mature as the British or Danish, even though it has developed quite rapidly during the last decade. It might be that we see a combination of young new-comers and women who have longer experience with eating organic food.

Household economy could be expected to be crucial for the level of organic consumption given the fact that these products generally are more expensive. However, our finding of an inverse relation between household income and frequency of organic consumption indicate a complexity beyond economic ability alone, and that other factors are more decisive for the likelihood of eating organic food. Higher levels of education, on the other hand, were associated with a higher likelihood of eating organic food compared with middle-levels of education, and so was being a student - even in the older age groups. Both education and income are strongly associated with better health in population studies. Our finding of lower household income may therefore be interpreted as an indicator of vulnerability with regard to health, while higher levels of education - particularly with increasing age, provides for robustness. The combination of higher education and lower income among the frequent consumers of organic food may point to a different value-orientation in this group.

Previous Norwegian surveys have reported higher levels of education among consumers with more frequent use of organic food and food produced without use of pesticides [29,59,62], while a regional survey did not find any association between length of education and likelihood of buying organic food [63]. Studies from other countries have also reported differing results regarding respondents' level of education and use of organic food [30,57,64,65]. Higher income was associated with a higher likelihood of buying organic food in one Norwegian study [63], while no difference with regard to income was reported in another [29]. A study of consumption of organic food in Denmark and Great Britain reported an increased propensity to buy organic food with higher 'social group', a composite indicator of educational level and income. However, in both countries, the highest propensity to buy organic food was found for the middle class households, while it was actually lower for the upper middle class [61]. Another Danish study also found that income explained very little of the purchasing behavior related to organic food [30].

Taken together, the lifestyle- and socio-economic characteristics of the frequent organic food consumers in the present study point to a complex phenomenon, involving diverse groups of women which go beyond narrowly defined 'consumer segments'. Some of the characteristics, such as regular exercise, lower BMI, and - in part - higher education indicate robustness with regard to a healthy lifestyle, while other characteristics, such as cigarette smoking and use of alcohol, and - in part - lower levels of education, may indicate vulnerability with regard to health of mother and child.

Our finding of a higher prevalence of frequent organic users in urban areas, is contrary to previous Norwegian studies that reported no difference between urban and rural areas [29,63], but in line with results from Denmark and Great Britain [60,66]. Further, our more nuanced finding that the highest level of organic consumption in the urban areas was followed by the most rural areas, with the lowest consumption in the two middle categories, is supported by other findings [67], and may be related to the possible 'idealistic' nature of organic consumption. Codron et al (2006) describe that a typical development for radical movements, such as those associated with organic food, is that when these products first enter the market, buyers are typically either local rural consumers, or urban consumers with higher income levels, including members of consumer associations and politically active movements sharing these values. In later phases, the constellations may change. These suggested lines of development may be relevant for Norway, where the market situation may still be described as not having reached maturity with regard to organic food - and we do find the highest levels of consumption among the contrasting groups: either urban or rural.

## Conclusions

Our results indicated that frequent use of organic food is a practice that is adopted among pregnant women across various 'groups' referring to the personal, lifestyle and socio-demographic variables investigated. This fits well with earlier observations that the consumption of organic food in the general Norwegian population has not been limited to special sub-groups, but rather quite widely distributed across socio-economic groups.

We did find certain characteristics to be more common among the frequent organic food consumers, but these characteristics were diverse and not necessarily associated with a healthy lifestyle. Lower levels of education and income as well as smoking and use of alcohol in pregnancy were less favorable factors in relation to health in our cohort. On the other hand, being lean and doing regular exercise are in line with health recommendations. A vegetarian diet may well be part of a healthy lifestyle. In conclusion, we found that in our material associations between socio-demographic and lifestyle variables and eating organic food were complex and could not be reduced to one single 'healthy lifestyle' orientation. However, given the relatively short period of time that organic food has been available to the majority of people in Norway, patterns of organic food consumption may keep changing as the market grows more mature. Characteristics of pregnant women who eat organic food may then change, possibly making differences between socio-demographic groups and the association to the different lifestyle factors more easily discernable. A methodological implication of the results is that personal and socio-economic characteristics should be regarded as important covariates in future studies of potential health outcomes related to organic food consumption during pregnancy.

## Competing interests

The authors declare that they have no competing interests.

## Authors' contributions

HMM initiated and was PI of the study. HT and HMM designed the study. HT performed the statistical analysis assisted by HS, ALB and MH. HT wrote the manuscript. ALB, GL, GR, MH and GHO participated in the design of the study and contributed to the manuscript. All authors read and approved the final manuscript.

## Pre-publication history

The pre-publication history for this paper can be accessed here:

http://www.biomedcentral.com/1471-2458/10/775/prepub
